# The Fight Against Cancer: Nitrobenzaldehyde as the Potential Warrior

**DOI:** 10.7759/cureus.2163

**Published:** 2018-02-06

**Authors:** Hira Saleem, Unzela Iqbal

**Affiliations:** 1 Dow Medical College, Dow University of Health Sciences (DUHS), Karachi, Pakistan

**Keywords:** cancer, photodynamic therapy, nitrobenzaldehyde

## Abstract

New milestones have been reached in oncology with the advent of a noninvasive, photodynamic therapy which aims to eradicate cancer cells rapidly. A chemical compound, Nitrobenzaldehyde, injected into the tumor, activates by ultraviolet (UV) light and disrupts the cancer cells' internal and external dynamics. This technique could be of enormous therapeutic value in destroying numerous cancer lines including breast, prostate, pancreatic cancers, etc., without causing unwanted systemic side effects.

## Editorial

We would like to shed light on a recent breakthrough in oncology, first reported by Matthew Gdovin, in his research, published in Seminars of Clinical Biology, 2017 [1]. It was demonstrated that a chemical compound, Nitrobenzaldehyde, when injected into the tumor, prior to a beam of ultraviolet (UV) light, eliminated cancer cells from the body within two hours. This noninvasive, photodynamic therapy was tested on mice with triple negative breast cancer, which normally has a devastating prognosis. The tumor was loaded with 1 mM of Nitrobenzaldehyde, followed by flash photolysis by UV light. Significant reductions were noted in tumor growth and volume, thereby increasing chances of survival. Also, no physical and behavioral side effects were observed.

Cancer cells normally have mechanisms which enable them to create an alkaline intracellular pH and an acidic extracellular pH with respect to noncancer cells, via the activation and expression of certain regulatory proteins [2]. An acidic external environment is favorable to thrive in hypoxic conditions and promotes their growth and metastasis [3]. It also provides resistance against chemotherapy and aids in angiogenesis derived from vascular endothelial growth factors [4]. Nitrobenzaldehyde functions as a caged H+ carrier which remains inactive unless exposed to UV light (200 nm to 410 nm) [5]. Upon exposure and subsequent activation, a proton particle is released from this molecule, which results in intracellular acidification and hence, apoptosis of tumors and cancerous cell lineages. Its superiority to other interventions, which reduce intracellular pH, lies in the fact that it easily diffuses inside the cell, is innocuous in nature, and promptly acidifies upon exposure to UV light. Moreover, since it is not cancer specific, it can be effectively used against breast, pancreatic, prostate cancers and even multidrug resistant cancers. Meanwhile, the surrounding healthy tissue remains unscathed. However, further research is required to investigate its effects on noncancerous cells.

The current cancer treatment protocols witness a myriad of limitations owing to the side effects of chemotherapy, radiotherapy, and surgical interventions. Chemotherapy can affect the normal dividing cells alongside cancerous cells, including those lining the digestive tract, hair, and bone marrow cells. This predisposes the patient to systemic illnesses including anemia, fatigue, hair loss, gastrointestinal complications, etc. Similarly, radiotherapy can induce tissue damage and produce genetic mutations. Comorbidities preclude patients from being potential candidates for surgery, so does the presence of tumors near vital structures. Formerly, photodynamic therapy worked on the principle of causing the release of reactive oxygen species (ROS) which inhibited tumor growth. However, a delicate balance was imperative as small levels of ROS facilitated cancer cell survival, growth, and metastasis. Moreover, oncogenesis was induced in the nearby noncancerous tissue.

Efforts are now being made to develop a photoactivated nanoparticle, which could target metastasized cells. It can also help surgeons remove tumors located in more critical positions such as the aorta, brainstem, or the spine. This novel photodynamic therapy is just one of the advancements, which has started to pave the road towards providing the most effective treatment option in the fight against cancer. However, much still needs to be done before cancer becomes a thing of the past.
